# Assessing and Mitigating the Risks for Polio Outbreaks in Polio-Free Countries — Africa, 2013–2014

**Published:** 2014-08-29

**Authors:** McKenzie Andre, Chris G. Wolff, Rudolf H. Tangermann, Paul Chenoweth, Graham Tallis, Jean Baptiste Kamgang, Steven G.F. Wassilak

**Affiliations:** 1 Global Immunization Division, Center for Global Health, CDC; 2 Polio Eradication Department, World Health Organization

Since 1988, when the Global Polio Eradication Initiative (GPEI)* began, the annual number of polio cases has decreased by >99% (1). Only three countries remain that have never interrupted wild poliovirus (WPV) transmission: Afghanistan, Nigeria, and Pakistan (1). Since 2001, outbreaks have occurred in 31 formerly polio-free counties in Africa, with outbreaks in 25 countries caused by WPV originating in Nigeria (2–4). After the declaration of the World Health Assembly of polio eradication as a programmatic emergency in 2012, efforts to identify areas at high risk for importation-associated outbreaks and to reduce that risk have been intensified (5). This report updates the 2013 assessment of the risk for outbreaks attributable to importation of poliovirus in 33 countries in Africa, using indicators of childhood susceptibility to poliovirus and proximity to countries currently affected by polio (6). From January 2013 to August 12, 2014, outbreaks occurred in five African countries. Four of the five (Cameroon, Equatorial Guinea, Ethiopia, and Somalia) have had recent transmission (cases within the previous 12 months). Based on the current risk assessment, 15 countries are considered to be at high risk for WPV outbreaks, five at moderate-to-high risk, seven at moderate risk, and six at low risk. In 15 of the 33 countries, less than half of the population resides in areas where surveillance performance indicators have met minimum targets (7). Enhanced, coordinated activities to raise childhood immunity are underway in 2014 to prevent additional WPV spread. Although substantial progress toward polio eradication has occurred in Nigeria, all African countries remain at risk for outbreaks as long as WPV continues to circulate anywhere on the continent.

## Current Outbreaks

Two multicountry outbreaks have occurred in Africa during 2013–2014 because of WPV imported from Nigeria; both are ongoing (4). The first outbreak, confirmed in May 2013 in the Horn of Africa, currently totals 222 cases: Somalia (198), Kenya (14), and Ethiopia (10). The first identified case was in a child in Somalia with onset in April 2013; subsequent cases were identified in eastern Kenya and in the Somali region of Ethiopia. Genomic sequence analysis of WPV isolates confirmed linkage of the cases and Nigeria as the source. The most recent cases were reported from the Puntland region of Somalia in June 2014. Transmission in Kenya appears to be interrupted, with onset of the latest case in July 2013; the latest case in Ethiopia had onset in January 2014.

A second outbreak in Central Africa (1), confirmed in October 2013, currently totals 14 cases: Cameroon (nine) and Equatorial Guinea (five). Genomic sequence analysis revealed that the outbreak virus originated in Nigeria and is most closely linked with a case in Chad in 2011, suggesting that this virus had been circulating undetected in the region for a substantial time before the first case was identified. The cases in Equatorial Guinea are linked to the Cameroon cases virologically and epidemiologically. The most recent case in Cameroon occurred in July 2014, and the most recent in Equatorial Guinea occurred in May 2014.

## Risk Assessment

In Nigeria, as of August 12, 2014, only five WPV cases have been reported in 2014, compared with 43 cases by the same date in 2013 and 83 cases by the same date in 2012. However, 25 African countries have experienced WPV importation outbreaks during 2009–2014. This analysis included those countries and eight selected neighboring countries. Childhood immunity indicators are derived from national coverage estimates for the third dose of oral poliovirus vaccine (OPV3) by age 12 months and the vaccination history of children aged 6–59 months who have been investigated for acute flaccid paralysis (AFP) with negative results for WPV. Reported values for each indicator are classified into risk tiers. Countries are considered to be at high risk for outbreaks if at least two of three of their immunity indicators fall in a high-risk tier (Table 1). Countries are considered to be at moderate-to-high risk for outbreaks if one of three childhood immunity indicators falls in a high-risk tier. Countries are considered to be at moderate risk for outbreaks if none of their population immunity risk criteria falls into a high-risk tier but one or more suggest moderate vulnerability. Countries are considered to be at low risk for outbreaks if all of their childhood immunity risk criteria suggest low vulnerability. The risk category for a country was increased one level if it shared a border with any country with recent transmission; this adjustment resulted in raising seven countries from the moderate-to-high–risk to the high-risk category and two countries from the low to the moderate risk category.

Other factors are taken into account when planning activities to mitigate risk for outbreaks after importation (Table 1). Surveillance performance indicators reflect the ability to detect cases quickly and thus limit spread of the virus. Country insecurity can disrupt routine immunization programs resulting in pockets of underimmunized children. The number of poliovirus importations for each country in the last 5 years reflects exposure history.

## Risk Mitigation Activities

### Immunization activities

Periodic preventive supplementary immunization activities (SIAs) take place nationally (national immunization days [NIDs]) and subnationally (subnational immunization days [SNIDs]) in polio-free countries, based on subnational immunization performance (Figure) to boost childhood immunity and limit vulnerability after WPV importation.

Of the 20 high-risk and moderate-to-high–risk countries, 17 implemented at least one SIA in 2012, and 18 implemented at least one SIA in 2013 (Table 2). During 2012, NIDs were conducted in seven of 15 high-risk countries and all five moderate-to-high risk countries; SNIDs were conducted in nine and four countries, respectively (Table 2). During 2013, NIDs were conducted in 11 of the high-risk countries and all five of the moderate-to-high risk countries. None of the five African countries with outbreaks in 2013 and 2014 had conducted nationwide polio SIAs in 2012, and none were planned in 2013 until after the first case in the countries was discovered. In 2014, every moderate-to-high and high-risk country is scheduled to have at least two nationwide campaigns, except for Angola, the Democratic Republic of the Congo, and Uganda.

### Strengthening surveillance

Strengthening AFP surveillance to promptly detect possible imported cases will speed response and limit the spread of WPV. The first major surveillance indicator is the rate of nonpolio acute flaccid paralysis (NPAFP) cases detected among children aged <15 years (the “NPAFP rate”). The target NPAFP rate is two or more cases per 100,000 children aged <15 years. A second major indicator, specimen adequacy, is the proportion of AFP cases for which two stool specimens were collected within 14 days of paralysis onset, ≥24 hours apart, arriving in good condition at an accredited laboratory. The target for stool specimen adequacy is ≥80%. Both indicators are analyzed at the national and provincial/state level (8).

Nine of the 15 high-risk countries had ≥80% of provinces with an NPAFP rate of two or more cases per 100,000 children aged <15 years (Table 1). Only six of the high-risk countries met the standard of having ≥80% of provinces meeting the target of ≥80% adequate stool specimens. In only six of the 15 high-risk countries was more than half of the population living in an area meeting both surveillance indicators. Two of the five moderate-to-high-risk countries had more than half of their population in areas meeting both surveillance indicators. Six of the seven moderate-risk and four of the six low risk countries had more than half of their population in areas meeting both surveillance indicators.

World Health Organization (WHO) regional offices and other GPEI partners conduct periodic activities to improve program performance and strengthen AFP surveillance. These include program assessments, AFP surveillance reviews, and surveillance training at national and subnational levels (Table 2). These activities are in addition to the development and testing of national emergency polio preparedness plans as recommended by the WHO African and Eastern Mediterranean regional certification commissions.

### Discussion

African countries continue to be at risk for WPV outbreaks, as proven by the two multicountry outbreaks during 2013–2014. Low immunity levels of children caused by chronic weaknesses in the delivery of immunization services puts countries at risk for continued spread of WPV after importation. Insecurity is a key factor in preventing children’s access to vaccines in Africa, including the complex humanitarian emergencies in Central African Republic, South Sudan, and Somalia. However, insecurity was not a factor in outbreaks in Cameroon and Equatorial Guinea.

GPEI partners recently formalized the way that risk assessments are conducted to better inform the planning of the international polio SIA calendar and provision of technical assistance. The current GPEI process of assessing risks in polio-affected WHO regions uses a consensus of the different approaches used by CDC, WHO, and the Institute for Disease Modeling based on the polio vaccination dose history of children with NPAFP as well as other factors. The review of country risk profiles in May 2014 was used by GPEI partners to plan the vaccination schedule for the second half of 2014, substantially increasing the number of planned campaigns in polio-free countries, and to plan focused technical assistance to enhance SIA effectiveness. The CDC-WHO risk tier approach presented in this report has essentially the same outcome as the formal, multi-agency approach conducted by GPEI partners in assessing susceptible children and is similar to the risk assessments conducted by WHO regional offices to set priorities for SIAs within regions and countries.

SIAs, by delivering large numbers of vaccinations in a short time, are the most effective way of rapidly boosting a population’s immune profile preventively or in response to an outbreak, especially where routine immunization of children is suboptimal. SIAs cannot fully correct for underlying problems in a country’s routine immunization program. Without recent WPV cases, many polio-free countries do not have recent experience in conducing national campaigns, and the campaigns suffer in quality. After the recent outbreaks, the quality of many of the initial SIAs in Africa was noted to be poor because of inadequate planning, supervision, and coordination (9). The effectiveness of SIAs can be improved by holding coordination workshops among Ministry of Health staff, community leaders, and local partners to develop “micro plans,” which are detailed implementation and local work plans that define the logistical needs, training, and supervision needed to conduct quality vaccination campaigns and identify repeatedly missed subpopulations.

The findings in this report are subject to at least four limitations. First, national coverage estimates are subject to overestimation. Second, annual NPAFP data for smaller countries can fluctuate substantially. Third, recall of dose histories might be inaccurate. Finally, surveillance might be limited or biased by imprecise population estimates.

In May 2014, in a coordinated international effort to reduce international exportation of WPV, WHO declared the recent international spread of WPV a public health emergency of international concern (10) and issued temporary recommendations under the International Health Regulations (IHR 2005). These recommendations include 1) ensuring that residents and long-term visitors, including adults, traveling from Cameroon, Equatorial Guinea, Pakistan, and Syria receive vaccination before international travel; 2) encouraging residents and long-term visitors, including adults, traveling from Afghanistan, Ethiopia, Iraq, Israel, Somalia, and Nigeria to receive vaccination before international travel; and 3) ensuring that such travelers are provided an International Certificate of Vaccination documenting vaccination status. The period for these recommendations was recently extended beyond August 3, 2014, with a reassessment to be conducted after 3 months.

The success of polio eradication efforts in Africa has led to a decrease in the number of countries with confirmed polio cases, from 20 in 2009 to three in 2012. In 2013, that number jumped to six, with more than 80% of the cases found outside of Nigeria, the one country on the continent in which polio is endemic. The events of the past 18 months show that all countries on the continent remain at risk for WPV outbreaks as long as circulation continues in Africa. To stop current outbreaks, prevent additional spread, and interrupt indigenous transmission in Nigeria, concerted efforts are needed to raise childhood immunity in the second half of 2014 and take the opportunity to interrupt all WPV transmission in Africa. Strengthening AFP surveillance in countries, regardless of a country’s risk status, will help identify poliovirus importations quickly and limit spread of disease. To achieve eradication, governments at national and subnational levels will need to remain politically committed, implement immunization activities as needed, strengthen AFP surveillance, and undertake other preparedness measures to lower the risk for polio outbreaks.

What is already known on this topic?

Only three countries have never interrupted wild poliovirus (WPV) transmission: Afghanistan, Nigeria, and Pakistan. In 2012, the World Health Assembly declared polio eradication a programmatic emergency for public health.

What is added by this report?

Using indicators of childhood susceptibility to poliovirus and proximity to countries currently affected by polio, 15 of the 33 countries in Africa without polio transmission since January 2013 were assessed to be at high risk for WPV outbreaks, five at moderate-to-high risk, seven at moderate risk, and six at low risk. Based on risk assessments, coordinated activities to raise childhood immunity and improve surveillance for acute flaccid paralysis (AFP) are underway in 2014 to prevent additional WPV spread.

What are the implications for public health practice?

Although substantial progress toward polio eradication has occurred in Nigeria, all African countries remain at risk for WPV outbreaks as long as WPV circulation continues on the continent. Global partners need to continue to identify and analyze different risk factors so that the proper technical and logistic support can mitigate the risk for ongoing transmission after poliovirus importation. To reach eradication, governments at the national and subnational levels will need to remain politically committed, improve AFP surveillance, implement immunization activities as needed, and undertake other preparedness measures to lower risk.

## Figures and Tables

**FIGURE:**
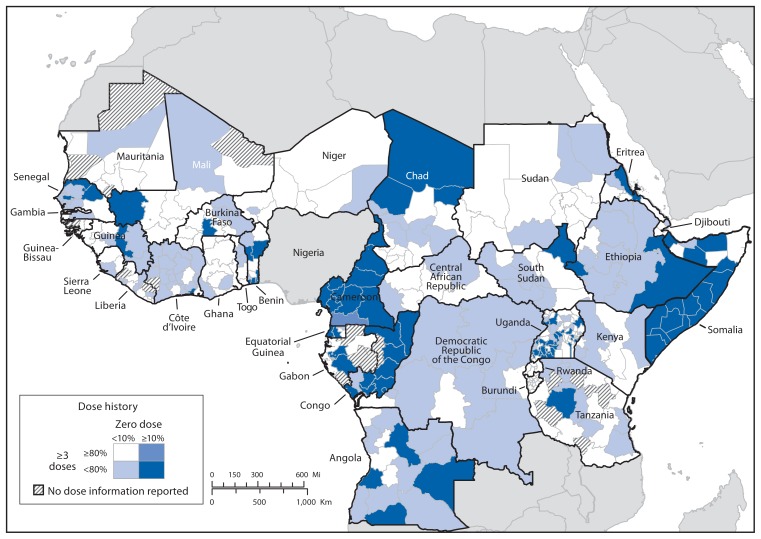
Oral poliovirus vaccine dose history among children aged 6–59 months with nonpolio acute flaccid paralysis in countries experiencing importations of wild poliovirus during 2009–2013 and selected neighboring countries — Africa, July 2013–June 2014

**TABLE 1:** Key risk indicators for 25 countries with wild poliovirus (WPV) cases during 2009–2014 and eight selected neighboring countries, by risk for transmission after an importation of WPV — Africa, 2014

	Childhood immunity indicators			Routine immunization indicators		Surveillance quality indicators			Other factors			
				
Risk level/Country	Risk tier: routine immunization with OPV3 national coverage*†	Risk tier: vaccine history ≥3 doses in children with NPAFP*,§	Risk tier: vaccine history zero doses in children with NPAFP¶	OPV3 national coverage (%)	% districts with ≥80% OPV3 coverage**	Risk tier for % provinces with ≥2 AFP cases per 100,000 children aged <15 yrs††	Risk tier for % of provinces with 80% of AFP stool specimen collection within 2 wks††	% of population residing in a province that meets both criteria	Areas of insecurity in the country that disrupt vaccine delivery§§	No. of WPV importations 2009–2013	Outbreak response countries (OR) and other priority countries (PC)	Borders with a country with WPV case in the last 12 months¶¶
**High risk**												
Benin	High	High	High	69	84	Mod	Low	59	No	0	PC	Yes
Cameroon	Mod	High	High	89	78	Low	High	36	Yes	3	OR	Yes
CAR	High	Low	Low	23	4	High	Low	35	Yes	2	PC	Yes
Chad	High	Mod	Low	48	60	Low	Mod	73	Yes	4	PC	Yes
Congo	High	High	High	69	73	Low	Mod	79	No	1	PC	Yes
Djibouti	Mod	High	Low	82	50	Low	Low	100	No	0		Yes
Equatorial Guinea	High	High	Low	3	6	High	High	0	No	0	OR	Yes
Ethiopia	High	High	Mod	72	50	Low	High	21	No	1	OR	Yes
Gabon	High	Low	Low	79	33	Low	High	0	No	1	PC	Yes
Guinea	High	High	Low	63	92	Low	Mod	38	No	2		No
Kenya	High	Low	Low	76	45	Low	Low	91	No	3		Yes
Niger	High	Low	Low	70	90	High	High	0	Yes	11	PC	Yes
Somalia	High	High	High	42	6	Low	Low	99	Yes	1	OR	Yes
South Sudan	High	Low	Low	45	23	High	Low	19	Yes	0		Yes
Uganda	High	High	Mod	78	83	Mod	Mod	46	No	0		No
**Moderate-to-high risk**												
Angola	Low	High	Low	93	66	Mod	Low	46	No	1	PC	No
Cote d’Ivoire	Mod	High	Low	88	99	Low	Mod	16	No	2	PC	No
DRC	High	Mod	Low	72	80	Low	Low	90	Yes	3	PC	No
Liberia	Mod	High	Low	89	87	Mod	Low	23	No	2		No
Mali	High	Low	Low	74	77	Low	Low	96	Yes	6	PC	No
**Moderate risk**												
Burkina Faso	Mod	Low	Low	88	100	Low	Low	95	No	1		No
Eritrea	Low	Low	Low	94	40	Mod	Low	65	No	1		Yes
Guinea-Bissau	Mod	Low	Low	80	91	Low	High	0	No	0		No
Mauritania	Mod	Low	Low	80	42	Low	Mod	61	No	1		No
Senegal	Low	Mod	Mod	92	32	Mod	Mod	54	No	3		No
Sudan	Low	Low	Low	93	93	Low	Low	100	Yes	0		Yes
Togo	Mod	Low	Low	84	95	Low	Low	58	No	2		No
**Low risk**												
Burundi	Low	Low	Low	96	93	High	Low	38	No	1		No
Gambia	Low	Low	Low	97	100	Low	Mod	67	No	0		No
Ghana	Low	Low	Low	90	75	High	Low	34	No	0		No
Rwanda	Low	Low	Low	98	100	Low	Low	100	No	0		No
Sierra Leone	Low	Low	Low	92	93	Mod	Low	79	No	1		No
Tanzania	Low	Low	Low	91	79	Mod	Low	66	No	0		No

**Abbreviations:** OPV3 = third dose of oral poliovirus vaccine; NPAFP = nonpolio acute flaccid paralysis; AFP = acute flaccid paralysis; CAR = Central African Republic; DRC = Democratic Republic of the Congo.

* Countries were considered to be at low risk at ≥90%, moderate risk at 80%–89%, and high risk at <80%.

† OPV3 coverage is based on World Health Organization (WHO)/United Nations Children’s Fund (UNICEF) estimates of the national vaccination coverage of the third dose of diphtheria-tetanus-pertussis (DTP3) for 2013.

§ Nonpolio acute flaccid paralysis database maintained by WHO.

¶ Countries were considered to be at low risk at <5%, moderate risk at 5%–10%, and high risk at >10%.

** Routine immunization administrative data as reported via WHO-UNICEF Joint Reporting Form, 2013, are based on use of DTP3 as proxy for OPV3 that does not count OPV doses administered during supplementary immunization activities.

†† Countries were considered to be at low risk at ≥80%, moderate risk at 50%–79%, and high risk at <50%.

§§ Areas with limitations in access and/or threats to the physical security of vaccination staff members.

¶¶ Nigeria, Cameroon, Ethiopia, Somalia, and Equatorial Guinea.

**TABLE 2:** Risk mitigation activities for 25 countries with wild poliovirus (WPV) cases during 2009–2014 and eight selected neighboring countries, by risk for transmission after an importation of WPV — Africa, 2012–2014

	Supplementary immunization activities: National and subnational immunization days (NID/SNID)				Surveillance strengthening activities: national program assessments (PA),* surveillance reviews (SR), or surveillance training activities (ST)	
		
Risk level/Country	Jan 2012–Dec 2012 (NID/SNID)	Jan 2013–Dec 2013 (NID/SNID)	Jan 2014–Jun 2014 (NID/SNID)	Jul 2014–Dec 2014 (NID/SNID) (Planned)	Jan 2013–Jun 2014	Jul 2014–Dec 2014 (Planned)
**High risk** †						
Benin	3/0	4/0	2/0	1/0	SR	
Cameroon	0/4	2/4	6/0	3/2	PA, ST	PA, ST
CAR	5/2	0/4	1/1	2/2		PA
Chad	3/10	4/8	2/1	1/3	SR, ST	PA
Congo	0/0	2/0	1/0	3/0		
Djibouti	1/0	3/0	0/1	2/0		
Equatorial Guinea	0/0	0/0	3/1	4/0		ST
Ethiopia	0/3	2/8	0/4	2/2	PA, ST	PA, ST
Gabon	0/0	0/0	1/0	3/0		ST
Guinea	4/1	3/0	0/0	2/0	SR	
Kenya	0/6	1/8	3/3	0/2	PA, SR, ST	PA, SR, ST
Niger	4/4	5/3	2/1	2/1	SR, PA	ST
Somalia	0/4	7/17	3/9	3/4	PA, ST	PA, ST
South Sudan	4/1	4/2	2/0	2/0		ST
Uganda	0/2	0/2	0/1	1/1		PA, SR
**Total at high risk**	**24/37**	**37/56**	**26/22**	**31/15**		
**Moderate-to-high risk**						
Angola	3/1	3/0	0/0	1/1		
Cote d’Ivoire	4/0	3/0	1/0	2/0	SR	
DRC	2/8	2/3	0/3	1/2	SR, ST	SR
Liberia	3/1	3/0	0/0	2/0	PA	
Mali	1/6	4/2	2/0	1/1	SR	
**Total at moderate-to-high risk**	**13/16**	**15/5**	**3/3**	**7/4**		
**Moderate risk**						
Burkina Faso	4/1	4/1	2/0	2/0	SR	
Eritrea	0/0	2/0	0/0	0/0		ST
Guinea-Bissau	1/0	2/0	0/0	2/0	SR	
Mauritania	3/0	2/0	0/0	2/0	SR	
Senegal	1/0	2/0	0/0	2/0	SR	
Sudan	3/1	2/2	1/1	1/0		
Togo	1/0	2/0	0/0	2/0		
**Total at moderate risk**	**13/2**	**16/3**	**3/1**	**11/0**		
**Low risk**						
Burundi	0/0	0/0	0/0	0/0		
Gambia	1/0	2/0	0/0	2/0		
Ghana	1/0	2/0	0/0	2/0	SR	
Rwanda	0/2	0/0	0/0	0/0		
Sierra Leone	2/1	4/0	0/0	2/0		
Tanzania	0/0	0/0	0/0	0/0		
**Total at low risk**	**4/3**	**8/0**	**0/0**	**6/0**		

**Abbreviations:** CAR = Central African Republic; DRC = Democratic Republic of the Congo.

* Program assessments include assessments conducted after the occurrence of a case of WPV infection or circulating vaccine-derived poliovirus infection and planned comprehensive immunization program reviews.

† Countries were considered to be at high risk for outbreaks in 2014 if two immunity indicators fell into a high-risk tier. Countries were considered to be at moderate-to-high risk for outbreaks if one of three immunity indicators fell into a high-risk tier. Countries were considered to be at moderate risk for outbreaks if any of the population immunity risk criteria suggested moderate vulnerability. A risk category for a country was increased one level if it shared a border with any country with recent transmission in the last 12 months.

## Footnotes

* The Global Polio Eradication Initiative is a public-private partnership led by national governments and led by the World Health Organization, Rotary International, CDC, and the United Nations Children’s Fund (UNICEF). Additional information available at http://www.polioeradication.org/aboutus.aspx#sthash.zicgwihb.dpuf.
